# Circulating Viral Antigenic Proteins and Inflammatory Biomarkers in Influenza and Respiratory Syncytial Virus Infections: Associations With Disease Severity and Transmission

**DOI:** 10.7759/cureus.100037

**Published:** 2025-12-24

**Authors:** Kailash Chandra, Ayan K Das, Bhumika Upadhyay, Azhar Uddin, Syed M Hussain, Farzana Islam, Yasir Alvi, Richa Gautam, Jaspreet Kaur, Sabina Khan, Rachna Tewari

**Affiliations:** 1 Department of Biochemistry, Hamdard Institute of Medical Sciences and Research, New Delhi, IND; 2 Department of Microbiology, Hamdard Institute of Medical Sciences and Research, New Delhi, IND; 3 Department of Community Medicine, Hamdard Institute of Medical Sciences and Research, New Delhi, IND; 4 Department of Community Medicine, Lady Hardinge Medical College, New Delhi, IND; 5 Department of Pathology, Hamdard Institute of Medical Sciences and Research, New Delhi, IND

**Keywords:** biomarkers, cytokine storm, disease severity, hemagglutinin, il-6, influenza virus, low- and middle-income countries, neuraminidase, respiratory syncytial virus, transmission

## Abstract

Influenza virus (INFV) and respiratory syncytial virus (RSV) remain major global causes of acute lower respiratory tract infections, leading to significant morbidity and mortality, particularly among infants, elderly adults, and individuals from low- and middle-income countries (LMICs). Despite advances in diagnostics and vaccines, early predictors of disease severity and transmission potential remain poorly defined.

A comprehensive literature search was conducted across PubMed, Scopus, and Web of Science databases for studies published between 2005 and 2025, focusing on viral biomarkers such as hemagglutinin (HA) and neuraminidase (NA) in influenza and host inflammatory biomarkers in both influenza and RSV infections. Eligible studies were included to evaluate the associations between these biomarkers and disease severity or transmission. A total of 41 studies were included, mainly from the USA and China, with smaller contributions from other countries. Both viral and host biomarkers were consistently linked to clinical outcomes. Elevated levels of circulating cytokines such as interleukin-6 (IL-6), interleukin-8 (IL-8), tumor necrosis factor-α (TNF-α), and C-reactive protein (CRP) were strongly associated with disease severity, intensive care admission, and mortality. High titres of anti-HA and anti-NA antibodies correlated with protection, reduced viral shedding, and limited transmission. Few studies integrated viral antigenic data with host inflammatory profiles, and evidence from LMICs, including India, remains limited.

Current evidence underscores the dual roles of viral surface antigens (HA/NA) and host inflammatory mediators in shaping disease outcomes and transmission dynamics in influenza and RSV infections. Integrative biomarker-based models combining viral antigenic, immunologic, and clinical parameters could enable early risk stratification, guide antiviral or vaccine strategies, and improve surveillance. Strengthening multicentric, longitudinal biomarker research, particularly in resource-limited settings, is essential to translate these findings into precision public health interventions.

## Introduction and background

Global burden of influenza virus (INFV) and respiratory syncytial virus (RSV) infections

INFV and RSV infections pose a considerable global health challenge, causing substantial morbidity and mortality annually, particularly in infants and the elderly. As per WHO estimates, seasonal influenza leads to three to five million serious infections and approximately three to 6.5 million deaths globally each year. The majority of such mortalities are reported from low- and middle-income countries (LMICs) like India [1]. Similarly, RSV, which is a leading cause of acute lower respiratory tract infections (LRTIs), causes more than 3.5 million serious infections leading to hospitalization and over a hundred thousand deaths among young children, mostly from LMICs. Some studies indicate that among older adults (over 60 years of age), the RSV-associated death rate is comparable or greater than that of INFV infection [2]. Severe infections requiring hospitalization create a notable economic strain on LMICs. A study revealed that the average cost of hospitalization for community-acquired pneumonia linked to INFV and RSV is considerably varied, amounting to around $305 in government-run hospitals and soaring to $1,210 in private hospitals in India [3]. This not only reflects the financial challenges faced by the affected population but also underscores the broader burden on the healthcare system of the nation.

Unlike temperate countries with a single seasonal peak, most often in winter, the Indian subcontinent (encompassing India, Pakistan, Nepal, Bangladesh, Sri Lanka, and neighboring countries) exhibits a complex pattern. The climatic heterogeneity, dense populations, and geographic latitude and monsoon seasonality are a few of the many factors that facilitate the year-round circulation of these viruses, with elevated activity during the monsoon season that may extend into the winter months (October to February) [4].

Within India, at least three different influenza peak patterns have been observed annually. The northern region of Jammu and Kashmir experiences a temperate winter peak (December-March), the densely populated Central region exhibits a tropical monsoon peak, and the southern coastal region encounters a late monsoon peak [5]. Within the central region, influenza A peaks during the monsoon and influenza B peaks during the post-monsoon autumn season. Overlapping this, the RSV infection peaks during the monsoon and continues till autumn/early winter. This creates a concurrent burden as both RSV and INFV are the two primary causes of severe acute respiratory infection (SARI) in young children [6]. These multifactorial complexities of a vast country like India warrant highly localized surveillance strategies.

Post-COVID-19 rebound studies in India (2021-2023) showed INFV and RSV resurgence, with influenza A(H3N2)/B-Victoria dominant in 2021 and A(H1N1)pdm09 in 2022. RSV accounted for 31.6% and INFV for 6.3% of pediatric SARI cases, with shifted seasonal peaks-earlier RSV and delayed influenza activity compared to pre-pandemic trends [6,7]. INFV and RSV infections present with similar symptoms, ranging from mild cold-like illness to severe lower respiratory disease. Common features include coryza, pharyngitis, high fever, myalgia, and fatigue. In infants and the elderly, both often progress to lower respiratory tract involvement. Hospitalization rates are high - 83.2% for RSV and 70% for influenza. Both trigger systemic inflammation with elevated C-reactive protein (CRP), erythrocyte sedimentation rate (ESR), mild hepatic dysfunction, and platelet changes. Coinfection in adults is linked to poorer outcomes [8-10]. As both these viruses impose a substantial burden on public health globally, early diagnosis, severity stratification, and transmission control become extremely important.

The present diagnostic modules for these viruses either lack sensitivity or are time-consuming and expensive for LMICs. Although highly sensitive assays like multiplex PCR can quickly identify the pathogen, they provide no clue regarding the patient's likely disease trajectory. There is an urgent necessity for identifying and validating circulating biomarkers like viral proteins or host inflammatory factors that can enable rapid risk stratification and guide targeted therapeutic interventions. Study of circulating viral antigenic proteins and host inflammatory biomarkers (e.g., interleukin-6 (IL-6), CRP) enables precise correlations with clinical outcomes, facilitating prognostic models, targeted therapies, and surveillance strategies. This review article aims to synthesize the current evidence by integrating findings from clinical, epidemiological, and immunological studies on the role of circulating viral antigenic proteins and inflammatory biomarkers in INFV and RSV infections and to identify knowledge gaps to guide future diagnostic, prognostic, and therapeutic strategies.

## Review

Influenza viruses (INFVs)

The INFV is an enveloped virus with segmented, single-stranded, negative-sense RNA and belongs to the *Orthomyxoviridae* family. Three out of the four major types, INFV A, B, and C, are known human pathogens. Other than man, the INFV-A also infects a wide range of mammals and birds, the INFV-B infects seals, and the INFV-C infects pigs. The newly discovered INFV-D mainly targets cattle and pigs. The virus may appear as spherical, elliptical, or filamentous particles. The spherical or elliptical particles, which are observed more frequently, have a diameter of 80 to 120 nm. The occasionally seen filamentous form, particularly significant for infectivity, and many even have a length exceeding 20 μm [11]. The INFV has a host-derived lipid envelope acquired during budding, containing glycoprotein spikes - hemagglutinin (HA) and neuraminidase (NA) - in a 4:1 ratio, along with ion channels (M2 in INFV-A, BM2 in INFV-B). INFV-B also carries an additional envelope protein, NB. The matrix protein M1 provides structural support, while INFV-C possesses a single major envelope protein, hemagglutinin-esterase-fusion (HEF), and a minor protein, CM2 [12]. INFV-A and B viruses have eight RNA segments (seven in C and D), enabling genetic reassortment. Each segment is coated with nucleoprotein (NP) and polymerases (PB1, PB2, PA), forming viral ribonucleoprotein complexes (vRNPs). The segments encode key proteins: PB2 (segment 1), PB1 and PB1-F2 (2), PA (3), HA (4), NP (5), NA (6; plus NMB in INFV-B), M1 and M2/BM2 (7), and nonstructural proteins NS1 and NS2 (8) [13,14].

HA, the main antigenic protein of INFV-A, mediates viral attachment to sialic acid receptors and membrane fusion, determining host tropism. There are 18 HA subtypes (H1-H18). NA, with 11 subtypes (N1-N11), cleaves sialic acid residues to prevent viral aggregation and aid release. Human infections mainly involve N1 and N2, while others occur in animals. NA is highly immunogenic; anti-NA antibodies reduce viral load, symptoms, and transmission by inhibiting virus release and promoting immune clearance [15,16]. INFV-A is classified by HA and NA subtypes, with 130 combinations identified - H1-H16/N1-N9 in aquatic birds, and H17N10/H18N11 in bats. Many avian subtypes can infect poultry and mammals, including humans [12]. INFV-B is classified by lineages, mainly B/Victoria and B/Yamagata, with both A subtypes and B lineages further divided into HA genetic clades and subclades [1].

Only a few influenza A subtypes circulate endemically in humans: H1N1 and H3N2 are seasonal, while H2N2 circulated from 1957 to 1968 before being replaced by H3N2 [12,14]. Pandemic A(H1N1) pdm09 emerged in 2009 from swine-origin viruses. Other subtypes, including avian-origin H5N1, H7N9, H5N6, and H9N2, infect humans sporadically, with limited human-to-human transmission; H5N1 and H7N9 have caused high mortality in humans [17]. INFV evolution relies on antigenic drift and antigenic shift. Antigenic drift involves point mutations in HA and NA, reducing antibody recognition and causing seasonal outbreaks, repeated infections, and partial vaccine escape. Antigenic shift is the sudden emergence of a novel strain with HA/NA unrelated to prior strains, usually via genetic reassortment in hosts co-infected with different INFV-A strains. Pigs, susceptible to both human and avian viruses, often mediate such reassortment, as seen in the 2009 H1N1 pandemic. Influenza B has a limited host range, reducing reassortment potential [18-21].

Respiratory syncytial virus (RSV)

RSV is an enveloped, non-segmented negative-strand RNA virus (00-350 nm; filamentous forms up to 20 μm) of the *Pneumoviridae* family. Its lipid envelope carries three glycoproteins - G (attachment), F (fusion), and SH (ion channel) - with G and F being key antigens for viral entry. The matrix protein (M) supports assembly and budding, while M2 proteins assist nucleocapsid function. The 15.2 kb genome encodes 11 proteins, including NS1 and NS2, which inhibit interferon responses and apoptosis, enhancing viral replication. The RNA is coated by nucleoprotein N and, with P and L proteins, forms the ribonucleoprotein complex [22,23]. RSV infects respiratory epithelial cells and immune cells by attachment via G protein to glycosaminoglycans/CX_3_CR1 receptor and fusion mediated by F protein, forming syncytia that spread the virus and damage airways. NS1 and NS2 proteins block immune responses by targeting MAVS, RIG-I, MDA5, and degrading STAT2, inhibiting interferon production. N and P proteins form inclusion bodies that sequester immune factors, while the G protein further evades immunity through its soluble form and CX_3_C motif, disrupting IFN signaling and leukocyte chemotaxis [23-26].

Transmission dynamics of INFV and RSV

Both INFV and RSV spread primarily via large respiratory droplets during close contact, and potentially aerosolized particles in enclosed settings [27]. RSV can survive for up to six hours on hard surfaces and for shorter durations on soft surfaces and can be transmitted easily through touch [28]. Though the spread of INFV through fomites on contaminated surfaces is possible, it is not well documented. Seasonal INFV-A and B spreads via close-range (~1-2 m) exposure to respiratory droplets (≥5 μm) and finer aerosols (<5 μm) expelled through coughing. Both viruses remain viable in airborne droplets for hours, irrespective of humidity in the environment [27]. The average incubation period for influenza is around two days (range one to four days), and for RSV is four to six days (range two to eight days). The INFV viral loads in the upper respiratory tract achieve their peak within one to two days of onset and then fall quickly (within approximately three days for influenza A, sometimes longer for B, three to eight days for RSV). Young children often shed both viruses for more than one week, while severely immunocompromised individuals may shed the virus for weeks to months [27]. Seasonal influenza is moderately transmissible, with a basic reproduction number (R0) around 1.3. RSV has higher transmissibility with an R0 of 3± 0.6 [29].

Household transmission is a critical factor in community spread of both INFV and RSV. The calculation of secondary attack rate (SAR) shows that children are the main source of household spread for both viruses. The estimated global SAR for RSV is considerably higher at 35.78% than that of the INFV (20.2%) [30,31]. Exposure to a school-going index patient has been associated with a statistically increased transmission risk compared to an adult index case. This may be augmented by the longer period of viral shedding by young infected cases compared to adults [27]. The size of the house and the number of members often determine the household spread.

Presence of viral proteins in host circulation

Several viral antigens are present in the host circulation during the infection. During proteolytic cleavage of RSV G protein, the soluble G fragment (sG) is released into the systemic circulation. The sG fragment contains a CX3C chemokine, which helps in immune evasion as discussed earlier [26]. The quantitative detection of sG may prove to be an effective prognostic biomarker.

During budding of new viral particles from the host cell, any structural proteins like HA, NA (for INFV), and F (RSV) may be released into the extracellular space. The host cells, when they undergo lysis, release internal contents, including these viral components, which may enter the local and systemic circulation [32]. Influenza is known to utilize host extracellular vesicles (EVs), such as exosomes, for the intercellular transfer of viral molecules. This transfer is critical for allowing immunomodulatory proteins like NS1 to reach the systemic circulation and inhibit the immune response. If NS1 is detected within plasma-derived exosomes, it would establish the infection's broad, systemic impact [33].

Quantification of soluble viral proteins in human plasma for routine assessment remains an analytical challenge. Current detection methods are indirect, measuring antibodies produced in the patient’s blood against these antigens. To truly establish the clinical relevance of circulating viral components, there is an urgent need to develop reliable, efficient assays that can directly measure the concentration of functional, soluble antigens (like free sG or NS1) in patient plasma.

Host inflammatory and immunological biomarkers

Severe influenza is often marked by an uncontrolled systemic inflammatory response, commonly known as a cytokine storm. This hyperinflammatory condition leads to tissue damage, widespread inflammation, and, ultimately, multiple organ dysfunction, which contributes to poor clinical outcomes and increased mortality [34-36].

Elevated levels of pro-inflammatory cytokines, especially IL-6 and tumor necrosis factor-alpha (TNF-α), are consistently observed in severe cases of influenza. These mediators play crucial roles during the acute inflammatory phase by driving fever, increasing vascular leakage, and activating immune cells. Numerous studies have demonstrated that serum IL-6 concentrations are significantly higher in patients requiring intensive care compared to those with milder forms of the disease. This suggests that IL-6 could serve as a potential predictive or prognostic biomarker [37-39].

Similarly, chemokines such as interleukin-8 (IL-8/CXCL8) and CXCL10 (IP-10) are markedly elevated in severe disease. IL-8 is a key neutrophil chemoattractant, while CXCL10 promotes T-cell trafficking to infection sites. Elevated levels of these chemokines, along with interferon-gamma (IFN-γ), reflect ongoing immune cell recruitment and correlate with the extent of lung injury and mortality risk [40-42].

Clinical laboratory markers remain essential tools for assessing systemic inflammation and disease progression. CRP, an IL-6-driven acute-phase reactant produced by the liver, is frequently elevated in severe influenza, including cases complicated by acute respiratory distress syndrome (ARDS). However, its non-specificity necessitates its interpretation alongside other indicators [43,44]. Ferritin levels, indicative of both iron metabolism and hyperinflammatory activity, are often markedly increased during cytokine storm syndromes and may serve as a surrogate marker of systemic immune activation [45]. Elevated D-dimer concentrations reflect coagulation activation and fibrinolysis, correlating with higher risks of thromboembolic events and mortality in severe influenza [46,47]. The neutrophil-to-lymphocyte ratio (NLR) has emerged as a simple yet robust predictor of disease severity and intensive care unit (ICU) admission. A high NLR indicates concurrent neutrophilia and lymphopenia, signifying an imbalance between innate hyperactivation and adaptive immune suppression [48,49].

Transcriptomic and proteomic response signatures

High-throughput omics technologies have deepened the understanding of host-pathogen interactions in influenza. Transcriptomic profiling of blood and respiratory samples has identified severity-associated gene signatures linked to antiviral defense, NF-κB and JAK-STAT pathways, and immune cell activation [50-52]. Such signatures help differentiate between adaptive, protective responses and maladaptive, pathogenic inflammation. Proteomic analyses, often integrated with transcriptomic data, have highlighted complement cascade activity, acute-phase proteins, and structural remodeling pathways as hallmarks of severe disease [53,54]. Comprehensive multi-omics integration enables the identification of complex molecular networks, supporting the development of predictive biomarkers and patient-specific therapeutic strategies.

Literature search, study selection, and outcomes

**Figure 1:**
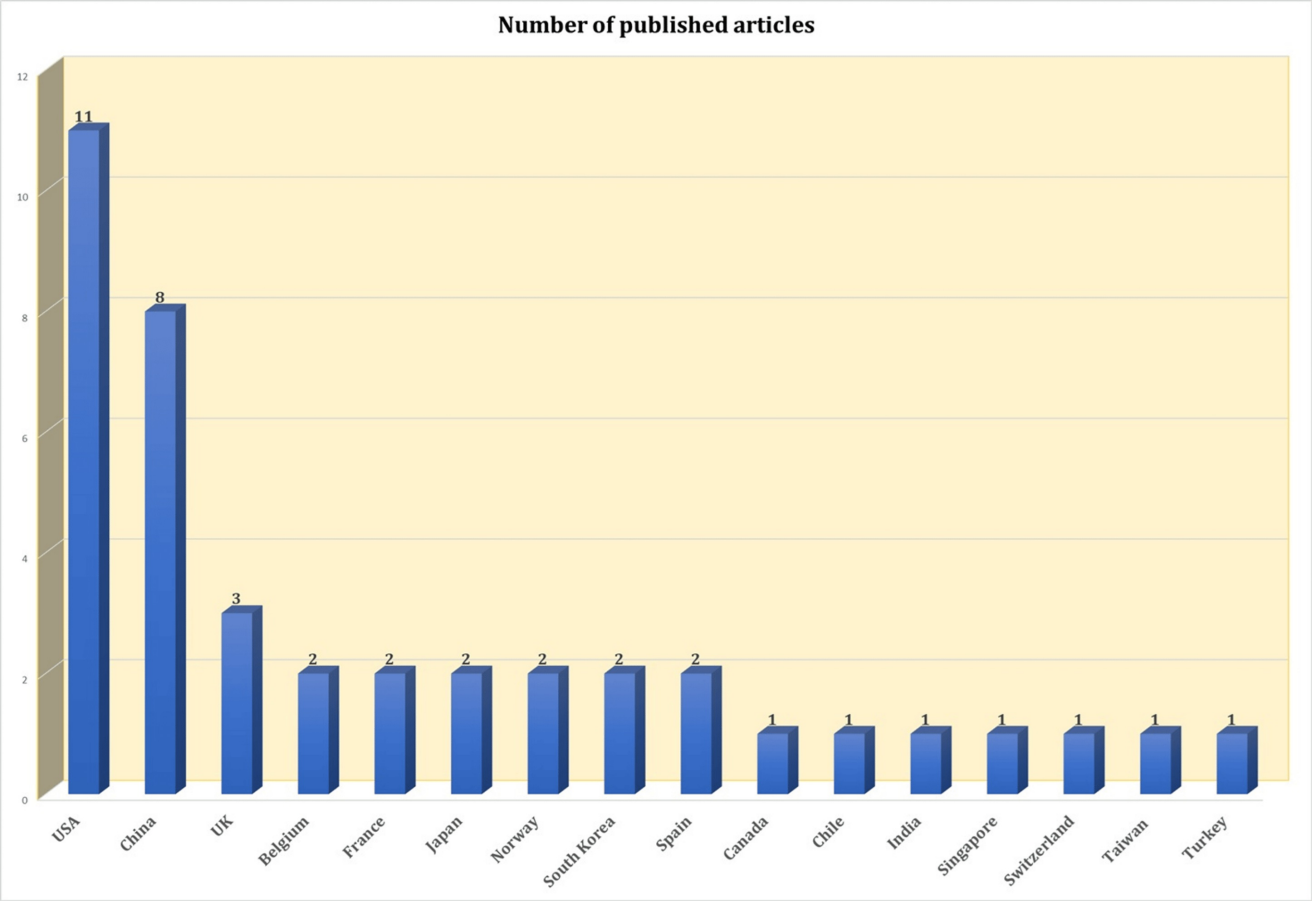
Country-wise distribution of published articles on hemagglutinin, neuraminidase, and inflammatory biomarkers in influenza and respiratory syncytial virus (RSV) infections between 2005 and 2025. References [55-94]

A thorough literature review was performed to identify studies examining circulating HA, NA, and inflammatory biomarkers in the context of INFV and RSV infections, with particular emphasis on their correlations with disease severity and transmission. We conducted a comprehensive search across databases, including PubMed, Scopus, and Web of Science, for articles published between 2005 and 2025 [55-94]. The search utilized a targeted strategy combining keywords and Boolean operators: "Circulating" AND ("Hemagglutinin" OR "Neuraminidase" OR "Biomarkers") AND "Inflammatory" AND ("Influenza" OR "RSV") AND ("Disease Severity" OR "Transmission"). Only English-language publications were included in this analysis. To ensure breadth and depth, both original research articles and review papers were considered, and the reference lists of pertinent studies were screened for additional relevant works. Eligible studies had to present data on circulating HA, NA, or inflammatory biomarkers in human populations infected with influenza or RSV, while also assessing their association with disease severity or transmission dynamics. This systematic search strategy allowed for a comprehensive aggregation of relevant literature over the past two decades, forming a solid foundation for this review. The distribution of published articles by country is illustrated in Figure 1.

The bar graph provides a detailed overview of the total number of research publications focused on INFV and RSV-associated biomarkers across various countries from 2005 to 2025. Notably, the United States emerges as the leader with an impressive total of 11 publications, reflecting its major contribution in this research domain. China follows as the second-highest contributor, with eight publications, indicating a growing emphasis on viral research and public health in the region. The United Kingdom ranks third with three publications. Several countries, including France, Belgium, Japan, Norway, South Korea, and Spain, each made moderate contributions, with two publications apiece. This suggests a steady, albeit limited, engagement in this specific area of study. Furthermore, Canada, Chile, India, Singapore, Switzerland, Taiwan, and Turkey each contributed one publication, demonstrating the participation of diverse countries, yet also highlighting the disparity in research outputs. Overall, this data reveals a clear dominance of high-income countries, particularly the United States, in influenza and RSV research. It underscores an urgent call for enhanced research efforts and resources from LMICs to create a more equitable global research landscape, ensuring that all nations can contribute to and benefit from advancements in public health.

The compiled studies clearly illustrate the interaction between viral surface antigens, specifically HA and NA, and host inflammatory biomarkers in determining disease severity and transmission potential for INFV and RSV infections (Table 1). Across various influenza studies, levels of anti-HA and anti-NA antibodies consistently emerged as important indicators of protection. Higher HA antibody levels, especially in children, were associated with reduced disease severity and better control of transmission. Additionally, immunity specific to NA provided broader protection across different viral lineages and was linked to lower viral shedding. Experimental and molecular investigations, such as those conducted by Sakai et al., Portela Catani et al., and Cortés et al., emphasized the importance of the functional balance between HA and NA in influencing viral movement, pathogenicity, and transmissibility. This underscores NA's often overlooked role in immune protection and vaccine development.

**Table 1:** Summary of studies (2005-2025) on circulating HA, NA, and inflammatory biomarkers in influenza and RSV: links to disease severity and transmission. HA: hemagglutinin; HAI: hemagglutination inhibition assay; GMT: geometric mean titer; RSV: respiratory syncytial virus; SARS-CoV-2: severe acute respiratory syndrome coronavirus 2; IL-6: interleukin-6; TNF-α: tumor necrosis factor alpha; IFN-γ: interferon gamma; IL-1β: interleukin-1 beta; IFN-α: interferon alpha; IFN-β: interferon beta; IFN-λ: interferon lambda; PRRs: pattern recognition receptors; RIG-I: retinoic acid-inducible gene I; NA: neuraminidase; ARI: acute respiratory infection; NLR: neutrophil-to-lymphocyte ratio; LMR: lymphocyte-to-monocyte ratio; PLT: platelet count; CRP: C-reactive protein; MPV: mean platelet volume; AUC: area under the curve; PLR: platelet-to-lymphocyte ratio; SII: systemic immune-inflammation index; ELISA: enzyme-linked immunosorbent assay; HI: hemagglutination inhibition; ELLA: enzyme-linked lectin assay; BALF: bronchoalveolar lavage fluid; MPO: myeloperoxidase; AIV: avian influenza virus; BPI: bactericidal/permeability-increasing protein; ELANE: neutrophil elastase; HPAI: highly pathogenic avian influenza; PCR: polymerase chain reaction; MPL-AF: monophosphoryl lipid A-adjuvant formulation; VLPs: virus-like particles; NGF: nerve growth factor; ERK: extracellular signal-regulated kinase; NP: nucleoprotein; AMs: alveolar macrophages; GM-CSF: granulocyte-macrophage colony-stimulating factor

Study (author, year)	Virus type	Study population/country	Age group	Biomarker(s) investigated	Key findings	Association with severity/transmission
Kondratiuk et al. (2025) [54]	Influenza A (H1N1pdm09, H3N2), Influenza B (Victoria & Yamagata)	700 patients (serosurvey); Poland	7 age groups: 0-4, 5-9, 10-14, 15-25, 26-44, 45-64, ≥65 yrs	Anti-HA antibodies (HAI assay); GMT; Protection rate (≥1:40)	Highest GMT (121.0) and protection (70%) for A/H3N2. Children 0-14 yrs had significantly higher GMT and seropositivity than adults for all antigens. Low vaccination rate (5.52%) → antibodies reflect natural infection.	High anti-HA titers (esp. in children) correlate with prior exposure; protective titers reduce severity; high seroprevalence in children implies role in community transmission.
Gambadauro et al. (2024 [55]	RSV, Influenza, SARS-CoV-2	Review of human studies (focus: newborns & pregnant women); International	Newborns (0-28 days), Pregnant women	Cytokines (IL-6, TNF-α, IFN-γ, IL-1β); Chemokines (CXCL8, CXCL10, CCL2/3/5); IFN-α/β/λ; PRRs (TLR3/7/8, RIG-I, MDA5)	RSV inhibits IFN-α/β but upregulates IL-6, TNF-α, CXCL8/10, CCL2/3/5. Influenza relies on IFN-I/TNF-α. SARS-CoV-2 in neonates → ↑IL-1β, IL-6, IFN-γ. Maternal infection alters fetal cytokine profile.	Dysregulated cytokines (↑IL-6, ↓IFN) → severe LRTI in neonates; maternal inflammation → fetal immune priming; high neonatal chemokines suggest early transmission risk.
Cortés et al. (2024) [56]	Influenza A/B	Human cohorts and vaccine trials; international; USA	Adults	NA-specific antibodies; HA-NA balance	NA immunity correlates with reduced disease; HA dominance limits NA response in vaccines.	Boosting NA antibodies reduces severity and transmission; low NA titer predicts higher viral shedding.
Huang et al. (2024) [57]	Influenza A and RSV	Hospitalized children with ARI; China	Children (<18 years)	Routine blood parameters (e.g., NLR, LMR, PLT); CRP	Elevated NLR/PLT in influenza vs. RSV; lower LMR in severe cases.	NLR and MPV/PLT predicted severity (AUC 0.6-0.7); differentiated viral etiology; high NLR linked to ICU need. Transmission not assessed.
Tang et al. (2024) [58]	H5N1 Influenza	Mice; China	N/A (Mice)	Immunity (Humoral, Cellular), Protection (Lethal Challenge)	A single-dose HA-based mRNA vaccine combined with a CpG adjuvant significantly enhanced and broadened both humoral and cellular immune responses in mice, resulting in complete protection against lethal challenge with heterologous H5N1 strains.	Presents an effective strategy for broadening vaccine protection against drifted/emerging strains, directly addressing prevention of severity and future transmission risk.
Okuyan et al. (2023) [59]	RSV	286 children with LRTI; Turkey	0-12 years	Systemic markers (NLR, PLR, SII, CRP)	RSV(+) had lower NLR, PLR, SII but higher CRP vs. RSV(−). AUC: CRP 0.869 (best predictor).	Lower NLR/PLR/SII in RSV(+) indicate viral vs. bacterial pattern; high CRP predicts severity. Children drive transmission.
Catani et al. (2023) [60]	Influenza B (Victoria & Yamagata lineages)	BALB/c mice (passive serum transfer model); Belgium	Adult mice	Anti-HA and anti-NA IgG (ELISA, HI, ELLA); NA-inhibiting antibodies; HA-inhibiting antibodies	Anti-NA sera (from both lineages) showed limited inter-lineage cross-reactivity but conferred superior protection vs. anti-HA sera against heterologous IBV challenge (e.g., Yamagata anti-NA protected better than anti-HA against Victoria strain).	NA immunity provides broader cross-lineage protection than HA; reduces morbidity/mortality in heterologous challenge; implies reduced transmission via lower viral shedding (indirect via protection).
Wiseman et al. (2020) [61]	RSV	Adults hospitalized with RSV vs. outpatient; UK (RESCEU consortium)	Adults (≥21 years)	Neutralizing antibodies; RSV-specific CD8+ T cells; airway IL-6, IL-8, MPO	Lower pre-infection neutralizing antibodies and RSV-specific IgA; higher airway IL-6/IL-8/MPO in severe cases; CD8+ T cells in BALF protective.	Low neutralizing antibodies correlated with susceptibility and severity (OR 2.2 for IL-6); high viral load associated with worse outcomes and potential transmission via prolonged shedding.
Forbester et al. (2020) [62]	Influenza A and RSV	Genetic analysis of patient cohorts; international; UK	Mixed (infants to adults)	Genetic variants in cytokines/chemokines (e.g., IL-6, IFN-γ, TNF-α); HA/NA interactions	Variants in cytokine genes associated with altered inflammatory responses; IL-6 elevations >200-fold in fatal RSV cases.	Cytokine variants predict severe LRTI (e.g., high IL-6 with lung damage); HA/NA balance influences transmission via receptor tropism.
Liu et al. (2020) [63]	Influenza A	Pediatric patients; China	Children (mean ~3 years)	Hub genes/proteins (e.g., LCN2, BPI, ELANE, MMP8); cytokines (e.g., IL-6)	Elevated BPI/MMP8 in severe/dead cases; decreased TCN1 in severe disease.	BPI and MMP8 predicted severe progression (AUC 0.7-0.8); linked to neutrophil-driven inflammation; no transmission focus.
Russier et al. (2020) [64]	H5N6 Avian Influenza Virus (AIV)	Mice (Animal model)/South Korea	N/A (Mice)	Cytokines/Chemokines (IL-6, TNF-α, IFN-γ, MCP-1), Histopathology (lung lesions), Body weight/Survival rate, Viral titer (lung)	A H5N6-E627K mutant virus (with a mutation in PB2) showed increased pathogenicity in mice compared to the wild-type virus. The mutant caused higher mortality, more severe lung lesions, and increased proinflammatory cytokines (IL-6, TNF-α).	The PB2 E627K mutation is a key determinant of high virulence in mammals, leading to increased disease severity (Severity/Virulence).
Song et al. (2019) [65]	Highly Pathogenic Avian Influenza (HPAI) H5N6 (Clade 2.3.4.4)	Chickens and Mice/China (Guangdong)	N/A (Animals)	Pathogenicity, Transmission, Viral Loads (brain, lung), Cytokines, Pattern Recognition Receptors (PRRs)	The H5N6 reassortant viruses showed high lethality and transmission in chickens. In mice, they caused mild to severe disease and demonstrated neuro-invasiveness (spread to the brain). High viral loads and up-regulation of immune factors correlated with severity.	Confirms high pathogenic potential in poultry and documents severe disease potential and neuro-invasiveness in mammals (Severity/Transmission).
Hijano et al. (2019) [66]	RSV and Influenza	Hospitalized children with LRTI; US	<5 years	Viral load (digital PCR); inflammatory markers (e.g., CRP)	Higher viral loads at presentation in symptomatic vs. asymptomatic; loads decreased post-antiviral (oseltamivir/ribavirin).	RSV loads positively correlated with symptoms/severity in ≤5 years; influenza loads associated with symptoms regardless of age; high loads predict transmission via shedding.
Dutta et al. 2019 [67]	Influenza	Influenza-infected patients; Assam, Northeast India	Not specified (adults/children inferred from context)	Pro- and anti-inflammatory cytokines (e.g., IL-6, TNF-α, IL-10)	Differential cytokine profiles observed in infected population; elevated pro-inflammatory cytokines in acute phase.	Differential cytokine expression may modulate disease severity; suggests potential for cytokine antagonists in severe cases. No direct transmission data.
Vázquez et al. (2019) [68]	RSV	Review of clinical studies; international; Chile	Infants/children	Airway cytokines (IL-6, IL-8, IFN-α, TSLP, IL-3, IL-33)	Severe LRTI: ↑IL-6, IL-8, IFN-α, TSLP, IL-3, IL-33 in NP/serum. Th2 bias in severe cases.	IL-8/IFN-α/TSLP predict hospitalization; high levels suggest prolonged shedding/transmission.
Kimoto et al. (2019) [69]	Avian Influenza Virus (AIV) (H7N9)	Mice (BALB/c)/Japan	N/A (Mice)	Vaccine Efficacy, Humoral Immunity (HI titer, IgG), Cellular Immunity (IFN-γ T-cells)	Evaluated an H7N9 vaccine formulated with a novel adjuvant (MPL-AF) compared to the standard alum adjuvant. MPL-AF significantly enhanced immunogenicity and protective efficacy against a lethal challenge, leading to better survival and less weight loss than alum.	The novel adjuvant formulation leads to a more effective vaccine, which is critical for prevention of severe disease caused by emerging H7N9 strains (Prevention/Severity).
Nguyen et al. (2019) [70]	Avian Influenza A (H9N2) virus	Chicken embryos and Mice/Belgium	N/A (Animals)	NS1 Gene Mutation (P42S), Virulence, Cytokine Production (IFN-β, IL-6, TNF-α), Viral Replication	Identified a natural mutation in the NS1 gene (P42S) in some H9N2 strains. The NS1-P42S mutant showed increased virulence in chicken embryos and enhanced replication in mammalian cells, but did not enhance mouse virulence in vivo.	The NS1-P42S mutation is an enhancer of virulence in birds, suggesting its role in transmission and potential to evolve for mammalian hosts (Transmission/Severity).
Van De Veerdonk et al. (2018) [71]	Influenza A (H1N1)pdm09 pandemic	Hospitalized adults (non-ICU)/Canada	Adults (>18 years, median age: 52)	Inflammatory Markers (Ferritin), Viral load (NP cycle threshold - Ct value)	Elevated serum ferritin levels (>= 300 μg/L) were associated with reduced viral shedding (higher Ct values) and worse clinical outcomes including need for oxygen, ICU transfer, and death.	Elevated ferritin levels at hospital admission are independently associated with influenza disease severity and poor clinical outcomes (Severity).
Elliott et al. (2018) [72]	Influenza A Virus (H3N2 Subtype)	Mice (BALB/c)/USA	N/A (Mice)	Humoral Immunity (Antibodies), Cellular Immunity (Antigen-specific Cytokine Response)	A multi-antigen Synthetic Micro-Consensus DNA Vaccine (pH3HA), delivered by intramuscular electroporation, induced broad humoral and cellular immunity against diverse seasonal H3N2 viruses and provided complete protection against lethal heterologous challenge.	The vaccine approach provides broad protection (Prevention) which would reduce severity and mortality caused by seasonal H3N2 drift variants (Prevention/Severity).
Sakai et al. (2017) [73]	Influenza A	In vitro/in vivo models (MDCK cells, mice); Japan	N/A (experimental)	HA and NA proteins (receptor-binding and cleavage activity)	HA-NA act as motile machinery for virus migration; balance required for efficient cell entry.	Imbalanced HA-NA reduces motility/transmission; optimal balance enhances pathogenicity and airborne spread.
Hu et al. (2017) [74]	Avian influenza A (H7N9) virus	BALB/c mice and specific-pathogen-free chickens (Animal models)/China/Taiwan	N/A (Mice and Chickens)	Virus-like particles (VLPs) (HA, NA, M1 proteins), HI serum titer, Antibodies (anti-NA, anti-M1), Splenic antigen-specific cytokines	Constructed H7N9 VLPs are multi-antigenic. VLP immunization generated elevated HI serum titer, NA/M1 antibodies, and increased antigen-specific cytokine production (cellular immunity).	H7N9 VLPs show desirable immunogenicity and are a candidate for vaccine development against H7N9 infection (Prevention/Transmission).
Choi et al. (2017) [75]	Influenza A virus (H1N1)	Murine macrophages (in vitro) and BALB/c Mice (in vivo) (Animal model)/South Korea/China	N/A (Mice/Cells)	Viral protein/mRNA expression (PB1, HA, M1, etc.), Cytokines (TNF-α, IL-6, IFN-β), IFN-related proteins (TBK1, STAT1, IRF3 phosphorylation), Natural Killer (NK) cell activity	Panax notoginseng root (PNR) extract significantly prevented viral infection in vitro and decreased mortality by 90% in mice. PNR enhanced antiviral IFN-mediated immune responses and NK cell activity.	PNR shows potential as an antiviral agent against influenza, reducing mortality (Severity/Treatment).
McBride et al. (2017) [76]	Influenza B virus (BR/08 virus)	Pharmacologically Immunosuppressed BALB/c mice (Animal model)/USA	N/A (Mice)	Monoclonal antibodies (mAbs) (anti-HA, anti-NA), Cytokines/Chemokines (IP-10, MCP-1), Virus titer (lungs)	Combination mAb therapy (anti-HA + anti-NA) protected mice from lethal infection and restricted virus spread; combination therapy led to higher IP-10 and MCP-1 expression in lungs.	mAb immunotherapy shows potential for treating influenza in immunocompromised hosts at high risk of severe disease (Severity/Prevention).
Garnier et al.(2016) [77]	Influenza Virus (H1N1)	Mice (In vivo model)/France	N/A (Mice)	B cell Memory, CD4+ T cell dependence, Antibody Affinity Maturation	Found that memory B cells generated after a primary influenza (H1N1) infection do not require cognate CD4+ T cell help to produce IgG antibodies upon a secondary, non-replicating challenge.	Sheds light on the mechanism of T cell-independent memory response, which is critical for rapid and sustained immunity (Prevention/Immunity).
Brown et al. (2015) [78]	RSV	Review of clinical/epidemiologic studies; international; USA	Infants/children	Neutrophil markers; cytokines (IL-8, IL-6, TNF-α); neurotrophins (NGF)	Elevated neutrophil activation, IL-8, and NGF in severe bronchiolitis; limited predictive biomarkers identified.	High IL-8/NGF predict ICU need; no single biomarker sufficient. High viral load implies transmission risk.
Johnstone et al. (2015) [79]	Influenza A Virus (H1N1pdm09)	Human Alveolar Epithelial Cells (A549) (In vitro)/Spain	N/A (Cells)	Proteomics (Protein Phosphorylation), Kinases (ERK), Viral Proteins (NP)	A quantitative phosphoproteomics approach identified numerous host proteins whose phosphorylation status changes upon infection. Key finding: the virus activates the host ERK signaling pathway, which is essential for efficient viral replication.	The study identifies the host ERK pathway as a vulnerability that the virus exploits to increase replication and severity. Inhibition of ERK severely impairs viral growth (Severity/Pathogenesis).
Mohn et al. (2015) [80]	Influenza A(H1N1)pdm09 pandemic	46 adult patients (hospitalized acute and convalescent)/Norway	Adults (>15 years)	Humoral Antibodies (HI, Microneutralization, IgG ELISA), T-cells (CD4+, CD8+ IFN-γ T-cells, TNF-α single-producing CD4+ T-cells)	Protective antibody responses increased with enhanced disease severity. Acute severe patients had higher levels of TNF-α single-producing CD4+ T-cells. Acute phase showed low cross-reactive CD8+ T-cells to internal antigens, while convalescent patients showed high CD4+ and CD8+ T-cells directed against conserved core antigens (NP, M).	Higher protective antibody responses and specific T-cell subsets are associated with the phase and severity of the disease (Severity/Immune response marker).
Tanner et al. (2014) [81]	Influenza A virus	Mice (C57BL/6)/Singapore	N/A (Mice)	Pulmonary Phospholipids (Alveolar and whole lung), Alveolar Macrophage Function	Infection caused significant changes in pulmonary phospholipid composition (a key component of lung surfactant), which persisted into the recovery phase. These changes were associated with impaired alveolar macrophage function (phagocytosis).	Alterations in lung lipid metabolism are a pathogenic mechanism contributing to lung injury and impaired host defense, thus increasing severity (Severity/Pathogenesis).
Guihot et al. (2014) [82]	Influenza A(H1N1) 2009 pandemic	34 critically ill, mechanically ventilated adults/France (Multicenter study)	Young to middle-aged adults (median age: 35 years)	Humoral Antibodies (HI, Microneutralization, ELISA), Cellular Immunity (T-cell responses, IFN-γ ELISpot), Cytokines (IL-6, IL-10), Viral RNA (viremia), Hemagglutinin mutation (222G)	Fatal fulminant cases had consistently low/negative serum HI antibodies and poor specific T-cell responses in peripheral blood. High plasma IL-6 and IL-10 correlated with severity. The negative serum serology was linked to antibody trapping in immune complexes in the lungs.	Negative HI serology ≤4 days after symptom onset predicts death from fulminant influenza (Severity).
Tabarani et al. (2013) [83]	RSV	851 children with LRTI; US	≤5 years	30-plex cytokines/chemokines in NP wash (IL-1β, IL-1RA, IL-6, IL-7, IL-8, TNF-α, IFN-α, CCL2/3/4, EGF, HGF)	Severe cases (ICU) had higher IL-1β, IL-1RA, IL-6, IL-7, TNF-α, IFN-α, CCL2/3/4, EGF, HGF vs. mild/moderate. RSV-A more severe.	Novel biomarkers (IL-1β, HGF, EGF) predict severity; high CCL2/3/4 imply shedding/transmission.
Davey et al. (2013) [84]	Influenza A(H1N1)pdm09	Hospitalized patients with confirmed infection; international (Singapore, Vietnam, UK)	Adults (mean ~40 years)	Inflammatory/coagulation markers (e.g., IL-6, CXCL8, sTNFR-1, D-dimer);	Baseline elevations in IL-6, CXCL8, sTNFR-1, and D-dimer in severe cases; strong predictive value for progression to hospitalization or death.	High IL-6 and CXCL8 levels predicted 2-3x higher risk of severe outcomes (e.g., ICU admission); no transmission data.
Turner et al. 2013 [85]	Influenza A (H3N2, X31 strain HA) + RSV (A2 strain F protein)	C57BL/6 mice, TLR4 −/− and +/+ mice/USA (University of Georgia)	6-8 weeks (adult mice)	Anti-HA and anti-F antibodies (ELISA titers); HA acid stability not directly measured but subunit vaccine context; viral lung titers post-challenge	Co-vaccination with HA + F or conjugated HA-F enhanced anti-HA immunity (higher ELISA titers vs. HA alone); reduced lung viral burden for both influenza (X31, PR8) and RSV (A2) upon challenge; F acts as molecular adjuvant via TLR4; no vaccine-enhanced disease	Positive association: F protein enhances HA-specific immunity and cross-protection, reducing severity (lower viral loads); potential for improved transmission control via dual vaccine, but HA stability not addressed as transmission factor
Richt et al. (2012) [86]	2009 pandemic H1N1 influenza virus (H1N1pdm09)	Hospitalized children with varying severity/Taiwan	Children (<18 years)	C-Reactive Protein (CRP), Lymphocyte count, Platelet count, Radiological Findings	Lower Lymphocyte count and higher CRP levels were significantly associated with severe clinical manifestations (e.g., ICU admission, respiratory failure, shock). These markers are valuable for predicting severe disease early on.	Lymphopenia and elevated CRP are associated with increased severity in pediatric H1N1pdm09 infection (Severity/Prognosis).
Fukushi et al. (2012) [87]	Highly Pathogenic Avian Influenza (HPAI) H5N1	Mice (Animal model)/China	N/A (Mice)	Cellular Immunity (CD8+ T-cells, NK cells, B-cells), Cytokines (IL-10, IFN-γ, IL-6, TNF-α), Viral load (lung)	A live attenuated cold-adapted (CA) H5N1 vaccine administered intranasally induced strong mucosal and systemic immunity. Immunization resulted in no weight loss or mortality after lethal challenge, unlike non-vaccinated mice. Protection was associated with robust cell-mediated immunity (CD8+ T-cells) and high IFN-γ production.	The CA H5N1 vaccine provides complete protection against HPAI H5N1, suggesting a reduced severity and transmission risk (Prevention).
Svindland et al. (2011) [88]	Influenza Virus	Guinea Pig Model/Norway	Pregnant sows and offspring	Transmission Routes (Aerosol, Direct Contact, Vertical)	Demonstrated aerosol and direct contact transmission of influenza between guinea pigs. Crucially, it found no vertical transmission of the virus from infected pregnant sows to their offspring, suggesting the placenta acts as an effective barrier.	Addresses a key transmission pathway (Vertical transmission) and finds it is not a risk in this animal model (Transmission).
Glinsky (2011) [89]	Influenza A(H1N1)pdm09	Critically ill patients; global genomic analysis; USA	Adults	HA mutations (e.g., D222G in receptor-binding site); inflammatory cytokines (e.g., IL-6)	Novel HA mutations associated with increased receptor-binding affinity; elevated IL-6 in severe cases.	HA mutations correlated with severe/fatal outcomes (e.g., higher lung tropism); indirect link to transmission via enhanced replication. Circulating HA not directly measured.
Bermejo-Martin et al. (2009) [90]	New variant of A/H1N1 pandemic influenza virus (nvH1N1)	Hospitalized (severe) and mild patients/Spain	Adults/Mixed (patients vs. controls)	Cytokines/Chemokines (IFN-γ, IL-8, IL-9, IL-17, IL-6, TNF-α, IL-15, IL-12p70, IP-10, MCP-1, MIP-1β), Antibodies (haemagglutination inhibition)	High systemic levels of Th1 and Th17 cytokines were exclusively found in hospitalized patients; Innate-immunity mediators (IP-10, MCP-1, MIP-1β) increased in all infected.	Th1 and Th17 hypercytokinemia is associated with severe pandemic infection (Severity).
Kim et al. (2008) [91]	Currently circulating human H1N1 influenza viruses (A/New Caledonia/20/99)	Pigs (Animal model for human influenza)/South Korea	N/A (Pigs)	Alveolar macrophages (AMs), TNF-α, IL-10, Antibody titers, CD8+ T lymphocytes (IFN-γ expression)	Depletion of AMs resulted in 40% mortality and more severe respiratory signs. Depleted pigs had lower TNF-α, higher IL-10, lower antibody titers, and fewer IFN-γ expressing CD8+ T lymphocytes.	Alveolar macrophages are essential for controlling H1N1 influenza viruses and their depletion leads to increased mortality (Severity).
Welliver et al. (2007) [92]	RSV and Influenza	Two study groups: surviving infants <12 months of age with either RSV or influenza virus infection. Infants had fatal LRTI. Postmortem lung tissue was obtained from infants with fatal RSV or influenza virus USA	<1 year	Cytokines/chemokines (e.g., IL-6, IL-8, MIP-1β); cytotoxic T cells (CD8+); viral antigen load	Extensive viral antigen in lung tissue; near absence of CD8+ lymphocytes and NK cells; high apoptotic markers (caspase-3); elevated nasopharyngeal IL-8 and MIP-1β in severe cases.	Inadequate adaptive immune response (low CD8+) and robust viral replication linked to fatal severity; no direct transmission link, but high viral load implies increased shedding.
Orson et al. (2006) [93]	Influenza	Mice (BALB/c)/USA	N/A (Mice)	Antibody Formation (Neutralizing Antibodies), Cytokines (IL-12, GM-CSF), Viral Titer (lung)	Aerosol delivery of a DNA vaccine encoding the HA antigen, when co-administered with plasmids for the cytokines IL-12 and GM-CSF, resulted in markedly increased neutralizing antibodies and complete protection against high-level virus proliferation.	Cytokine-enhanced aerosol DNA vaccination is a robust strategy for prevention, which would reduce transmission and severity across new influenza strains (Prevention).
Hassa et al. (2005) [94]	Influenza A Virus (H5N1)	Human Lung Epithelial Cells (A549) (In vitro); Switzerland	N/A (Cells)	Apoptosis (Caspase Activation), Viral Proteins (NS1), Cytokines (TNF-α)	H5N1 virus was shown to be a potent inducer of apoptosis in infected A549 cells, much more so than a low-pathogenic H1N1 strain. The NS1 protein plays a critical role in promoting apoptosis by sensitizing cells to apoptotic stimuli like TNF-α.	Enhanced apoptosis is a virulence determinant of H5N1, contributing to severe lung damage and disease severity (Severity/Pathogenesis).

Inflammatory biomarkers such as IL-6, IL-8, TNF-α, CRP, and NLRs are strongly linked to disease progression, especially in cases of severe LRTIs caused by influenza and RSV. Elevated levels of IL-6 and CXCL8 consistently predict hospitalization and mortality in influenza patients. Studies on RSV, including those by Tabarani et al., Okuyan et al., and Wiseman et al., have shown that increased airway cytokine responses (IL-6, IL-8, TSLP) and disrupted immune profiles are associated with greater disease severity and prolonged viral shedding. This suggests an increased potential for transmission, particularly in children. Overall, these findings highlight that both the viral antigenic characteristics (such as HA and NA) and the host's inflammatory responses influence the clinical outcomes and epidemiological patterns of influenza and RSV infections. This insight points to dual targets for enhancing diagnostics, prognostics, and vaccine strategies.

The summary of circulating HA, NA, and inflammatory biomarkers related to disease severity and transmission is presented in Table 2. Numerous studies involving both humans and animals indicate that humoral responses to HA and NA, along with viral load and innate inflammatory markers - particularly IL-6, IL-8, and neutrophil indices - are consistently linked to the clinical severity of influenza and RSV. Pre-existing antibodies targeting HA and mucosal IgA are associated with a reduced susceptibility to infection. Emerging evidence also suggests that antibodies that inhibit NA may provide broader cross-lineage protection and potentially decrease virus shedding. However, there is a lack of prospective data directly connecting serial biomarker changes to measured transmission rates. The variability in testing assays and limited data from LMICs, including India, hinders the generalizability of these findings. To effectively translate biomarkers into interventions that reduce both disease severity and transmission, standardized longitudinal cohort studies integrating virology, serology (for HA and NA), and host immunogenomics are essential.

**Table 2:** Summary of circulating hemagglutinin, neuraminidase, and inflammatory biomarkers in disease severity and transmission. NA: neuraminidase; HA: hemagglutinin; IL-6: interleukin-6; IL-8: interleukin-8; IP-10: interferon gamma-induced protein 10; ICU: intensive care unit; CCL2/3/4: C-C chemokine ligand 2, 3, and 4; NLR: neutrophil-to-lymphocyte ratio; CRP: C-reactive protein; CD8+: cluster of differentiation 8 positive T cells

Biomarker class	Representative finding	Association with severity	Association with transmission
Anti-HA (HI titers) [14]	High HI = protection; low HI early predicts fatality [H1N1]	↑ HI → ↓ severity	Pre-existing HI reduces susceptibility → lowers transmission
Anti-NA/NA-inhibiting Abs [16]	NA immunity offers broader protection; low NA → high shedding	↑ NA → ↓ severity	Higher NA immunity → reduced shedding
Viral load [34]	High viral load → worse outcomes	↑ viral load → ↑ severity	↑ viral load → ↑ shedding
Inflammatory cytokines (IL-6, TNF-α, IL-8, IP-10) [36]	Elevated in severe cases	Strong severity marker; predicts ICU/death	Chemokines (CCL2/3/4, IP-10) link to shedding
Neutrophil indices (NLR, CRP, ferritin) [41,43,47]	NLR, CRP, and ferritin correlate with severity	Useful for triage/severity prediction	Not routinely linked to transmission
Cellular immunity (CD8+, mucosal IgA) [15]	Pre-existing immunity → mild disease	Robust immunity → mild disease	Mucosal IgA reduces susceptibility/spread

Research gaps and future directions

Despite extensive global research linking circulating viral antigens and inflammatory biomarkers to influenza and RSV severity, several critical knowledge gaps remain:

Limited Longitudinal and Population-Based Data

Most available studies are cross-sectional or based on small hospital cohorts, which restricts our understanding of changes in biomarker levels over time, immune durability, and post-infection transmission dynamics. There is a lack of longitudinal surveillance that integrates serological, clinical, and molecular parameters, particularly in LMICs.

Underrepresentation of Developing Countries, Including India

The majority of published data originated from Europe, the Americas, and East Asia. While Indian studies provide preliminary cytokine profiling in influenza-infected individuals from Assam, they are limited by small sample sizes, a lack of standardized biomarker assays, and the absence of parallel virological or antigenic analysis (e.g., HA/NA characterization). There is a noticeable scarcity of RSV biomarker data from India, with few or no reports correlating circulating cytokines, chemokines, or viral load to disease severity.

Inadequate Integration of Viral Antigenic and Host Inflammatory Markers

Most studies concentrate on either the viral immune response (anti-HA/NA antibodies) or inflammatory cytokine patterns, but very few integrate both datasets. This integration is essential to understand how antigenic variation influences host inflammatory responses and clinical outcomes. The relationship between HA-NA balance and cytokine dysregulation in co-infections (e.g., influenza-RSV) has not been sufficiently explored.

Scarcity of Pediatric and Community-Level Transmission Data

Although children are recognized as key transmitters of infections, as demonstrated in various serosurveys, large-scale studies that quantify how biomarker profiles correlate with viral shedding, contagious periods, and household transmission are limited. This gap is particularly critical in India, where overcrowding and low vaccination rates increase the risk of community transmission.

Lack of Standardized Biomarker Thresholds for Disease Severity

While markers such as IL-6, CRP, and NLR show predictive value, there is no consensus on cut-off values or assay standardization across studies. This absence of uniformity makes it challenging to apply findings effectively in clinical or public health contexts.

Vaccine and Antigenic Data Gaps

Data from India regarding circulating HA and NA variants, seroconversion rates, and vaccine-induced immunity are limited.

In India, influenza is a recurring seasonal and post-monsoon infection, with variable regional dominance of H1N1 and H3N2 strains. However, there are few serological and cytokine correlation studies available. The limited studies, such as Dutta et al. (2019), conducted in Assam, indicate elevated levels of pro-inflammatory cytokines (IL-6, TNF-α) during acute influenza infections, but they lack follow-up data on disease severity or transmission outcomes. For RSV, there is a high rate of infant hospitalizations during the winter seasons, yet systematic biomarker-based studies are absent. There is an urgent need for integrated multicentric research that combines viral genomics (such as HA/NA profiling), immune biomarker quantification, and clinical severity assessment.

## Conclusions

INFV and RSV continue to present significant global health and economic challenges, with overlapping seasonal peaks and similar mechanisms for immune activation and disease progression. Over the past two decades, evidence has shown that circulating viral proteins - especially HA and NA - along with host inflammatory mediators such as IL-6, IL-8, TNF-α, CRP, and neutrophil indices, play crucial roles in determining disease severity, potential for transmission, and clinical outcomes. High levels of anti-HA and anti-NA antibodies provide protection and reduce the amount of virus shed, while hyperinflammatory responses are characteristic of severe and fatal cases.

Despite recent advancements, significant research gaps remain in the field. Most studies are conducted in high-income countries, which means that LMICs, including India, are often underrepresented in biomarker-based surveillance and clinical correlation studies. Additionally, the limited integration of viral antigen data with host immune signatures, along with the lack of standardized biomarker thresholds, impedes clinical translation. To address these issues, future research should focus on multicentric, longitudinal studies that combine viral genomics, antigenic profiling, host immune transcriptomics, and clinical outcomes. Such integrative approaches will help identify reliable biomarkers for disease prognosis and transmission control. This, in turn, will inform targeted vaccination strategies, therapeutic interventions, and public health preparedness in various epidemiological contexts.

## References

[1] (2025). Influenza (seasonal). https://www.who.int/news-room/fact-sheets/detail/influenza-(seasonal).

[2] Landi SN, Garofalo DC, Reimbaeva M (2024). Hospitalization following outpatient diagnosis of respiratory syncytial virus in adults. JAMA Netw Open.

[3] Prabhakaran AO, Amarchand R, Kanungo S (2025). Resource utilisation and cost of hospitalisation with community-acquired pneumonia among older adults in India, 2018-2020. BMJ Public Health.

[4] Nisar N, Badar N, Safdar I (2025). Assessing influenza activity variations in the Asian region during the pre- and post-pandemic period (2017-2023). PLoS One.

[5] Chadha MS, Potdar VA, Saha S (2015). Dynamics of Influenza Seasonality at Sub-Regional Levels in India and Implications for Vaccination Timing. PLoS One.

[6] Khan T, Das RS, Jaiswal A (2025). Epidemiology and surveillance of influenza, RSV and SARS-CoV-2 in children admitted with severe acute respiratory infection in West bengal, India from 2022 to 2023. BMC Infect Dis.

[7] Potdar V, Vijay N, Mukhopadhyay L (2023). Pan-India influenza-like illness (ILI) and severe acute respiratory infection (SARI) surveillance: epidemiological, clinical and genomic analysis. Front Public Health.

[8] Devadiga S, Varamballi P, Shetty U, Mukhopadhyay C, Jayaram A (2025). Respiratory viral infections in hospitalized adults: a comparative clinico-laboratory study of RSV, HMPV, and influenza. Virol J.

[9] Recto CG, Fourati S, Khellaf M (2024). Respiratory syncytial virus vs influenza virus infection: mortality and morbidity comparison over 7 epidemic seasons in an elderly population. J Infect Dis.

[10] Zhang Y, Zhao J, Zou X (2020). Severity of influenza virus and respiratory syncytial virus coinfections in hospitalized adult patients. J Clin Virol.

[11] Dadonaite B, Vijayakrishnan S, Fodor E, Bhella D, Hutchinson EC (2016). Filamentous influenza viruses. J Gen Virol.

[12] Liang Y (2023). Pathogenicity and virulence of influenza. Virulence.

[13] Fodor E, Te Velthuis AJ (2020). Structure and function of the influenza virus transcription and replication machinery. Cold Spring Harb Perspect Med.

[14] Krammer F, Smith GJD, Fouchier RAM (2018). Influenza. Nat Rev Dis Primers.

[15] Crowe JE Jr (2019). Antibody determinants of influenza immunity. J Infect Dis.

[16] Giurgea LT, Morens DM, Taubenberger JK (2020). Influenza neuraminidase: a neglected protein and its potential for a better influenza vaccine. Vaccines.

[17] Wang WH, Erazo EM, Ishcol MR, Lin CY, Assavalapsakul W, Thitithanyanont A, Wang SF (2020). Virus-induced pathogenesis, vaccine development, and diagnosis of novel H7N9 avian influenza A virus in humans: a systemic literature review. J Int Med Res.

[18] Chen Z, Bancej C, Lee L, Champredon D (2022). Antigenic drift and epidemiological severity of seasonal influenza in Canada. Sci Rep.

[19] Khurana S, Hahn M, Coyle EM (2019). Repeat vaccination reduces antibody affinity maturation across different influenza vaccine platforms in humans. Nat Commun.

[20] Wang Y, Tang CY, Wan XF (2022). Antigenic characterization of influenza and SARS-CoV-2 viruses. Anal Bioanal Chem.

[21] Gong X, Hu M, Chen W (2021). Reassortment network of influenza A virus. Front Microbiol.

[22] Talukdar SN, Mehedi M (2022). Respiratory syncytial virus. IntechOpen.

[23] Ouyang Y, Liao H, Hu Y, Luo K, Hu S, Zhu H (2022). Innate immune evasion by human respiratory syncytial virus. Front Microbiol.

[24] Raiden S, Sananez I, Remes-Lenicov F (2017). Respiratory syncytial virus (RSV) infects CD4+ T cells: frequency of circulating CD4+ RSV+ T cells as a marker of disease severity in young children. J Infect Dis.

[25] Cadena-Cruz C, Villarreal Camacho JL, De Ávila-Arias M, Hurtado-Gomez L, Rodriguez A, San-Juan-Vergara H (2023). Respiratory syncytial virus entry mechanism in host cells: a general overview. Mol Microbiol.

[26] Meineke R, Agac A, Knittler MC (2024). Respiratory syncytial virus glycoprotein G impedes CX3CR1-activation by CX3CL1 and monocyte function. npj Viruses.

[27] Killingley B, Nguyen‐Van‐Tam J (2013). Routes of influenza transmission. Influenza Other Respir Viruses.

[28] Kaler J, Hussain A, Patel K, Hernandez T, Ray S (2023). Respiratory syncytial virus: a comprehensive review of transmission, pathophysiology, and manifestation. Cureus.

[29] Baraldi E, Checcucci Lisi G, Costantino C (2022). RSV disease in infants and young children: can we see a brighter future?. Hum Vaccin Immunother.

[30] Duan Q, Pan J, Zheng L (2025). Global outbreaks of respiratory syncytial virus infections from 1960 to 2025: a systematic review and meta-analysis. EClinicalMedicine.

[31] Savage R, Whelan M, Johnson I (2011). Assessing secondary attack rates among household contacts at the beginning of the influenza A (H1N1) pandemic in Ontario, Canada, April-June 2009: a prospective, observational study. BMC Public Health.

[32] Carvajal JJ, Avellaneda AM, Salazar-Ardiles C, Maya JE, Kalergis AM, Lay MK (2019). Host components contributing to respiratory syncytial virus pathogenesis. Front Immunol.

[33] Hong Y, Truong AD, Vu TH (2022). Exosomes from H5N1 avian influenza virus-infected chickens regulate antiviral immune responses of chicken immune cells. Dev Comp Immunol.

[34] de Jong MD, Simmons CP, Thanh TT (2006). Fatal outcome of human influenza A (H5N1) is associated with high viral load and hypercytokinemia. Nat Med.

[35] Short KR, Kroeze EJ, Fouchier RA, Kuiken T (2014). Pathogenesis of influenza-induced acute respiratory distress syndrome. Lancet Infect Dis.

[36] Teijaro JR, Walsh KB, Rice S, Rosen H, Oldstone MB (2014). Mapping the innate signaling cascade essential for cytokine storm during influenza virus infection. Proc Natl Acad Sci U S A.

[37] Langnau C, Rohlfing AK, Gekeler S (2021). Platelet activation and plasma levels of furin are associated with prognosis of patients with coronary artery disease and COVID-19. Arterioscler Thromb Vasc Biol.

[38] Kalinowski A, Ueki I, Min-Oo G (2014). EGFR activation suppresses respiratory virus-induced IRF1-dependent CXCL10 production. Am J Physiol Lung Cell Mol Physiol.

[39] Novak T, Crawford JC, Hahn G (2023). Transcriptomic profiles of multiple organ dysfunction syndrome phenotypes in pediatric critical influenza. Front Immunol.

[40] Zhang N, Bao YJ, Tong AH (2018). Whole transcriptome analysis reveals differential gene expression profile reflecting macrophage polarization in response to influenza A H5N1 virus infection. BMC Med Genomics.

[41] Pepys MB, Hirschfield GM (2003). C-reactive protein: a critical update. J Clin Invest.

[42] Levinson T, Wasserman A, Shenhar-Tsarfaty S (2023). Comparative analysis of CRP as a biomarker of the inflammatory response intensity among common viral infections affecting the lungs: COVID-19 versus influenza A, influenza B and respiratory syncytial virus. Clin Exp Med.

[43] Kernan KF, Carcillo JA (2017). Hyperferritinemia and inflammation. Int Immunol.

[44] Goeijenbier M, van Wissen M, van de Weg C (2012). Review: viral infections and mechanisms of thrombosis and bleeding. J Med Virol.

[45] Yang Y, Tang H (2016). Aberrant coagulation causes a hyper-inflammatory response in severe influenza pneumonia. Cell Mol Immunol.

[46] Chen L, Yin Z, Zhou D (2024). Lymphocyte and neutrophil count combined with intestinal bacteria abundance predict the severity of COVID-19. Microbiol Spectr.

[47] Faria SS, Fernandes PC Jr, Silva MJ (2016). The neutrophil-to-lymphocyte ratio: a narrative review. Ecancermedicalscience.

[48] Zhai Y, Franco LM, Atmar RL (2015). Host transcriptional response to influenza and other acute respiratory viral infections-a prospective cohort study. PLoS Pathog.

[49] Tisoncik-Go J, Gasper DJ, Kyle JE (2016). Integrated omics analysis of pathogenic host responses during pandemic H1N1 influenza virus infection: the crucial role of lipid metabolism. Cell Host Microbe.

[50] Zhou A, Dong X, Liu M, Tang B (2021). Comprehensive transcriptomic analysis identifies novel antiviral factors against influenza A virus infection. Front Immunol.

[51] Shen S, Li J, Hilchey S (2016). Ion-current-based temporal proteomic profiling of influenza-A-virus-infected mouse lungs revealed underlying mechanisms of altered integrity of the lung microvascular barrier. J Proteome Res.

[52] Powell JD, Waters KM (2017). Influenza-omics and the host response: recent advances and future prospects. Pathogens.

[53] Blanco-Melo D, Nilsson-Payant BE, Liu WC (2020). Imbalanced host response to SARS-CoV-2 drives development of COVID-19. Cell.

[54] Kondratiuk K, Masny A, Poznańska A (2025). Incidence of circulating antibodies against hemagglutinin of influenza viruses in epidemic season 2023/2024 in Poland. Biomolecules.

[55] Gambadauro A, Galletta F, Li Pomi A, Manti S, Piedimonte G (2024). Immune response to respiratory viral infections. Int J Mol Sci.

[56] Cortés G, Ustyugova I, Farrell T (2024). Boosting neuraminidase immunity in the presence of hemagglutinin with the next generation of influenza vaccines. NPJ Vaccines.

[57] Huang L, Ye C, Zhou R, Ji Z (2024). Diagnostic value of routine blood tests in differentiating between SARS-CoV-2, influenza A, and RSV infections in hospitalized children: a retrospective study. BMC Pediatr.

[58] Tang P, Cui E, Cheng J (2024). A ferritin nanoparticle vaccine based on the hemagglutinin extracellular domain of swine influenza A (H1N1) virus elicits protective immune responses in mice and pigs. Front Immunol.

[59] Okuyan O, Elgormus Y, Dumur S, Sayili U, Uzun H (2023). New generation of systemic inflammatory markers for respiratory syncytial virus infection in children. Viruses.

[60] Portela Catani JP, Ysenbaert T, Smet A, Vuylsteke M, Vogel TU, Saelens X (2023). Anti-neuraminidase and anti-hemagglutinin immune serum can confer inter-lineage cross protection against recent influenza B. PLoS One.

[61] Wiseman DJ, Thwaites RS, Drysdale SB, Janet S, Donaldson GC, Wedzicha JA, Openshaw PJ (2020). Immunological and inflammatory biomarkers of susceptibility and severity in adult respiratory syncytial virus infections. J Infect Dis.

[62] Forbester JL, Humphreys IR (2021). Genetic influences on viral-induced cytokine responses in the lung. Mucosal Immunol.

[63] Liu S, Huang Z, Deng X, Zou X, Li H, Mu S, Cao B (2021). Identification of key candidate biomarkers for severe influenza infection by integrated bioinformatical analysis and initial clinical validation. J Cell Mol Med.

[64] Russier M, Yang G, Briard B (2020). Hemagglutinin stability regulates H1N1 influenza virus replication and pathogenicity in mice by modulating type I interferon responses in dendritic cells. J Virol.

[65] Song Y, Li W, Wu W (2019). Phylogeny, pathogenicity, transmission, and host immune responses of four H5N6 avian influenza viruses in chickens and mice. Viruses.

[66] Hijano DR, Brazelton de Cardenas J, Maron G (2019). Clinical correlation of influenza and respiratory syncytial virus load measured by digital PCR. PLoS One.

[67] Dutta M, Dutta P, Medhi S, Borkakoty B, Biswas D (2019). Immune response during influenza virus infection among the population of Assam, Northeast India. Indian J Med Microbiol.

[68] Vázquez Y, González L, Noguera L, González PA, Riedel CA, Bertrand P, Bueno SM (2019). Cytokines in the respiratory airway as biomarkers of severity and prognosis for respiratory syncytial virus infection: an update. Front Immunol.

[69] Kimoto T, Kim H, Sakai S, Takahashi E, Kido H (2019). Oral vaccination with influenza hemagglutinin combined with human pulmonary surfactant-mimicking synthetic adjuvant SF-10 induces efficient local and systemic immunity compared with nasal and subcutaneous vaccination and provides protective immunity in mice. Vaccine.

[70] Nguyen GT, Rauw F, Steensels M, Ingrao F, Bonfante F, Davidson I, Lambrecht B (2019). Study of the underlying mechanisms and consequences of pathogenicity differences between two in vitro selected G1-H9N2 clones originating from a single isolate. Vet Res.

[71] Van De Veerdonk F Jr, Dewi I, Cunha C (2018). 967. Inhibition of host neuraminidase increases susceptibility to invasive pulmonary aspergillosis. Open Forum Infect Dis.

[72] Elliott ST, Keaton AA, Chu JD (2018). A synthetic micro-consensus DNA vaccine generates comprehensive influenza A H3N2 immunity and protects mice against lethal challenge by multiple H3N2 viruses. Hum Gene Ther.

[73] Sakai T, Nishimura SI, Naito T, Saito M (2017). Influenza A virus hemagglutinin and neuraminidase act as novel motile machinery. Sci Rep.

[74] Hu CJ, Chien CY, Liu MT (2017). Multi-antigen avian influenza a (H7N9) virus-like particles: particulate characterizations and immunogenicity evaluation in murine and avian models. BMC Biotechnol.

[75] Choi JG, Jin YH, Lee H, Oh TW, Yim NH, Cho WK, Ma JY (2017). Protective effect of Panax notoginseng root water extract against influenza a virus infection by enhancing antiviral interferon-mediated immune responses and natural killer cell activity. Front Immunol.

[76] McBride JM, Lim JJ, Burgess T (2017). Phase 2 randomized trial of the safety and efficacy of MHAA4549A, a broadly neutralizing monoclonal antibody, in a human influenza A virus challenge model. Antimicrob Agents Chemother.

[77] Garnier A, Hamieh M, Drouet A (2016). Artificial antigen-presenting cells expressing HLA class II molecules as an effective tool for amplifying human specific memory CD4(+) T cells. Immunol Cell Biol.

[78] Brown PM, Schneeberger DL, Piedimonte G (2015). Biomarkers of respiratory syncytial virus (RSV) infection: specific neutrophil and cytokine levels provide increased accuracy in predicting disease severity. Paediatr Respir Rev.

[79] Johnstone C, Lorente E, Barriga A (2015). The viral transcription group determines the HLA class I cellular immune response against human respiratory syncytial virus. Mol Cell Proteomics.

[80] Mohn KG, Cox RJ, Tunheim G (2015). Immune responses in acute and convalescent patients with mild, moderate and severe disease during the 2009 influenza pandemic in Norway. PLoS One.

[81] Tanner LB, Chng C, Guan XL, Lei Z, Rozen SG, Wenk MR (2014). Lipidomics identifies a requirement for peroxisomal function during influenza virus replication. J Lipid Res.

[82] Guihot A, Luyt CE, Parrot A (2014). Low titers of serum antibodies inhibiting hemagglutination predict fatal fulminant influenza A(H1N1) 2009 infection. Am J Respir Crit Care Med.

[83] Tabarani CM, Bonville CA, Suryadevara M (2013). Novel inflammatory markers, clinical risk factors and virus type associated with severe respiratory syncytial virus infection. Pediatr Infect Dis J.

[84] Davey RT Jr, Lynfield R, Dwyer DE (2013). The association between serum biomarkers and disease outcome in influenza A(H1N1)pdm09 virus infection: results of two international observational cohort studies. PLoS One.

[85] Turner TM, Jones LP, Tompkins SM, Tripp RA (2013). A novel influenza virus hemagglutinin-respiratory syncytial virus (RSV) fusion protein subunit vaccine against influenza and RSV. J Virol.

[86] Richt JA, Rockx B, Ma W (2012). Recently emerged swine influenza A virus (H2N3) causes severe pneumonia in cynomolgus macaques. PLoS One.

[87] Fukushi M, Yamashita M, Miyoshi-Akiyama T, Kubo S, Yamamoto K, Kudo K (2012). Laninamivir octanoate and artificial surfactant combination therapy significantly increases survival of mice infected with lethal influenza H1N1 Virus. PLoS One.

[88] Svindland SC, Jul-Larsen Å, Pathirana R (2012). The mucosal and systemic immune responses elicited by a chitosan-adjuvanted intranasal influenza H5N1 vaccine. Influenza Other Respir Viruses.

[89] Glinsky GV (2010). Genomic analysis of pandemic (H1N1) 2009 reveals association of increasing disease severity with emergence of novel hemagglutinin mutations. Cell Cycle.

[90] Bermejo-Martin JF, Ortiz de Lejarazu R, Pumarola T (2009). Th1 and Th17 hypercytokinemia as early host response signature in severe pandemic influenza. Crit Care.

[91] Kim HM, Lee YW, Lee KJ (2008). Alveolar macrophages are indispensable for controlling influenza viruses in lungs of pigs. J Virol.

[92] Welliver TP, Garofalo RP, Hosakote Y (2007). Severe human lower respiratory tract illness caused by respiratory syncytial virus and influenza virus is characterized by the absence of pulmonary cytotoxic lymphocyte responses. J Infect Dis.

[93] Orson FM, Kinsey BM, Densmore CL, Nguyen T, Wu Y, Mbawuike IN, Wyde PR (2006). Protection against influenza infection by cytokine-enhanced aerosol genetic immunization. J Gene Med.

[94] Hassa PO, Haenni SS, Buerki C (2005). Acetylation of poly(ADP-ribose) polymerase-1 by p300/CREB-binding protein regulates coactivation of NF-kappaB-dependent transcription. J Biol Chem.

